# Application of the BACs-on-Beads assay for the prenatal diagnosis of chromosomal abnormalities in Quanzhou, China

**DOI:** 10.1186/s12884-021-03589-9

**Published:** 2021-01-28

**Authors:** Jianlong Zhuang, Chunnuan Chen, Yuying Jiang, Qi Luo, Shuhong Zeng, Chunling Lv, Yuanbai Wang, Wanyu Fu

**Affiliations:** 1 Prenatal Diagnosis Center, Quanzhou Women’s and Children’s Hospital, Quanzhou, Fujian 362000 People’s Republic of China; 2 Department of Neurosurgery, The Second Affiliated Hospital of Fujian Medical University, Quanzhou, Fujian 362000 People’s Republic of China; 3 Department of Public Health for Women and Children, Quanzhou Women’s and Children’s Hospital, Quanzhou, Fujian 362000 People’s Republic of China; 4 Zhejiang Biosan Technology Co., Ltd, Hangzhou, Zhejiang 310000 People’s Republic of China

**Keywords:** Prenatal diagnosis, BoBs assay, Karyotyping, Microdeletion/microduplication

## Abstract

**Background:**

An increasing number of techniques have been used for prenatal diagnosis of genetic abnormalities. Our initial objective was to explore the value of the BACs-on-Beads (BoBs) assay for the prenatal diagnosis of aneuploidies and microdeletion/microduplication syndromes in Quanzhou, Southeast China.

**Methods:**

A total of 1409 pregnant women with high-risk factors for chromosomal abnormalities admitted to Quanzhou Women’s and Children’s Hospital were enrolled in this study. BoBs assays and karyotype analyses were conducted for all subjects. Subsequently, chromosome microarray analysis (CMA) or fluorescence in situ hybridization (FISH) was performed to validate the findings.

**Results:**

In this study, karyotype analysis and BoBs assay failed in 4 cases, and 2 cases, respectively. A total of 1403 cases were successfully analyzed, with success rates of 99.72% (1405/1409) and 99.85% (1407/1409) for karyotype analysis and Bobs assay, respectively. BoBs assay rapidly detected chromosomal aneuploidies in line with the karyotyping data. Additionally, 23 cases of microdeletions/microduplications were detected by BoBs assay but missed by karyotyping, including 22q11.2 microdeletions/microduplications, 5p15.32p15.33 microdeletion, Xp22.31 microdeletions/microduplications, Xq27.3 microdeletion, and Yp11.2 and Yq11.22q11.222 microduplication. In comparison with karyotyping, fewer mosaicisms were identified by BoBs assay. A high detection rate of chromosomal abnormalities was observed in the high-risk group during noninvasive prenatal testing (NIPT) (41.72%) and the abnormal ultrasound group (13.43%).

**Conclusions:**

BoBs assay can be used for the rapid and efficient prenatal diagnosis of common aneuploidies and microdeletion/microduplication syndromes. Moreover, the combined use of BoBs assay and karyotyping in prenatal diagnosis may allow for a more effective detection of chromosomal abnormalities.

**Supplementary Information:**

The online version contains supplementary material available at 10.1186/s12884-021-03589-9.

## Background

Birth defects account for a high proportion of infant mortality and morbidity worldwide [1]. The prevalence of birth defects is approximately 5.6% in China, with chromosomal abnormalities being the most important etiologic factor for birth defects [2]. With the liberalization of the second child policy in China, the numbers of pregnant women with advanced maternal age is increasing, which may lead to a high prevalence of fetal chromosomal abnormalities. Karyotyping is the “gold standard” for the prenatal diagnosis of chromosomal abnormalities. However, cell culture takes a long time (At least 7 days) and displays low resolution (Greater than 5 Mb in size) [3]. Microdeletion and microduplication syndromes refer to a series of genetic diseases caused by microchromosomal aberrations, which ranging from 10^3^ to 10^6^ bp [4]. To date, more than 300 microdeletion and microduplication syndromes have been reported worldwide, with incidence rates ranging from 1/4000 to 1/50000 [5]. Patients with microdeletion and microduplication syndromes exhibit complex clinical manifestations, including abnormal growth and development, mental retardation, distinctive facial features, internal organ deformities, endocrine abnormalities and abnormal mental behavior. FISH (Fluorescence in situ hybridization), QF-PCR (Quantitative fluorescence PCR), BoBs, and CMA (Chromosome microarray analysis) are all diagnostic tools that can detect chromosomal microdeletions and microduplications, with high throughput detection and shorter reporting times. BoBs assay can be used for the rapid detection of common aneuploidies (13, 18, 21, X, and Y) and microdeletion syndromes (DiGeorge, Williams-Beuren, Prader-Willi, Angelman, Smith-Magenis, Wolf-Hirschhorn, Cri-du-Chat, Langer-Giedion, and Miller-Dieker syndromes) [6, 7]. Furthermore, BoBs assay can be used to diagnose chromosomal abnormalities and microdeletions within 30 h [8].

In this study, a total of 1409 pregnant women with high risk factors were enrolled for prenatal diagnosis, BoBs assay was performed to explore its application value in terms of detecting aneuploidies and microdeletion/microduplication syndromes in comparison with karyotyping in Quanzhou, China. This study also provides available data regarding the importance of prenatal diagnosis in the prevention and control of birth defects, and is of great significance in further supporting the application value of BoBs assay in prenatal diagnosis.

## Methods

### Subjects

A total of 1409 pregnant women with high-risk factors for chromosomal abnormalities admitted at Quanzhou Women’s and Children’s Hospital from January 2016 to December 2019 were enrolled in this study. The indications for prenatal genetic evaluation were advanced maternal age (> 35 years), high-risk NIPT results, abnormal ultrasound, high-risk serological screening results, and adverse pregnancy history. The subjects were divided into six groups, including an advanced maternal age group (*n* = 190), a high-risk NIPT results group (*n* = 163), an abnormal ultrasound group (*n* = 134), a high-risk serological screening results group (*n* = 480), an adverse pregnancy history group (*n* = 159) and a group of patients with two or more types of high-risk factors (*n* = 283). All of the participants signed an informed consent. Approval was obtained from the institutional ethics committee of Quanzhou Women’s and Children’s Hospital (2020No.12 and 2020No.31).

### Conventional karyotype analysis

Approximately 30 ml of amniotic fluid was obtained by amniocentesis, of which 20 ml was used for cell culture and 10 ml for BoBs detection. After centrifugation of the amniotic fluid, the suspension was inoculated in an amniotic fluid culture medium and cultured at 37 °C for 7–10 days. Amniocytes were harvested by trypsinization and the chromosomal preparation was performed using a SinochromeChromprepII automatic chromosome harvesting system (Shanghai Lechen Biotechnology Co., Ltd.). Additionally, G-band karyotype analysis was performed after staining with Giemsa. Subsequently, 30 metaphase cells were observed for each case, and 5 karyotypes were analyzed. C-banding was conducted when necessary.

### DNA extraction

The subjects’ DNA was extracted from uncultured amniotic fluid cells for BoBs assay and further SNP array verification. DNA extraction of amniotic fluid was performed using a QIAamp DNA blood Kit (QIAGEN, Germany), following the kit’s handbook (www.qiagen.com).

### BACs-on-Beads assay

The prenatal BoBs kit was used to detect common aneuploidies (13, 18, 21, X, and Y), and 9 common microdeletion syndromes according to the manufacturer’s instructions (PerkinElmer, Wallac, Turku, Finland) (Table 1). For this purpose, 50-250 ng of genomic DNA was labelled with enzyme-linked biotin-deoxynucleoside triphosphate. The labelled product was purified, and hybridized with normal DNA from reference males and females with BoBs probes and subjected to fluorescent incubation and mycotin in elution. The fluorescence signals were measured using a Luminex 200 platform and the results analyzed by the BoBsoft 1.0 software (PerkinElmer, Wallac, Turku, Finland). The results were described by comparing the data with female (Red line) and male (Blue line) controls. The ratio of the probe and its fluorescence intensity are presented in numerical and graphical forms. Samples with microdeletions/microduplications deviated from the normal ratio of 1.0. Regions with ratios ranging from 1.3 to 1.4 were deemed as duplications; meanwhile those with ratios between 0.6 and 0.8 were considered as deletions.

**Table 1:** The BAC probes distribution in BoBs assay

Syndrome	Target region	Number of probes
Trisomy 13	13q13.3-13q21.2	5
Trisomy 18	18p11.32-18q22.1	5
Trisomy 21	21q22.11-21q22.3	5
Trisomy X	Xp22.31-Xp27.3	5
Trisomy Y	Ypll.2-Yqll.23	5
Wolf-Hirschhorn	4p16.3	5
Cri du Chat	5p15.2-5p15.3	8
Williams-Beuren	7q11.2	5
Langer-Giedion	8q23-8q24	7
Prader-Willi/Angelman	15q11-15q12	7
Miller-Dieker	17p13.3	6
Smith-Magenis	17p11.2	4
DiGeorgeI	22q11.2	4
DiGeorgeII	10p14	4

### SNP chromosome microarray detection

A SNP chromosome microarray (AffymetrixCytoScan™ 750 K) was performed to verify samples with microdeletions/microduplications detected by BoBs assay. A total of 250 ng of genomic DNA was digested with the NspI enzyme, the ends were filled with ligase, and genomic DNA was amplified by polymerase chain reaction (PCR). After the product was purified and prepared for quantitative detection, the fragmentation reaction was carried out to generate fragments of approximately 25-125 bp, which were then labelled with biotin and hybridized with the probe on the chip. The hybridized chip was washed and stained, then analyzed after the fluorescent signal was scanned. The Chromosome Analysis Suite (ChAS) v4.0 software was used to analyze the fluorescence signal in order to determine the sample’s genome-wide variation of the sample. The pathogenicity of copy number variations was interpreted with reference to DGV (Database of Genomics Variations, http://dgv.tcag.ca/dgv), OMIM (Online Mendelian Inheritance in Man, https://omim.org/), DECIPHER (https://decipher.sanger.ac.uk/), and PubMed (https://www.ncbi.nlm.nih.gov/pubmed/), along with other databases.

### Fluorescence in situ hybridization analysis

Fluorescence in situ hybridization was performed on chromosomes 13, 18, 21, 22, X and Y using probes (Beijing GP Medical Technology Co., Ltd., Beijing, China) according to the manufacturer’s protocol. One hundred amniotic cells were counted and analyzed by fluorescence microscopy (Olympus, Tokyo, Japan) to determine the occurrence of chromosome aberrations.

### Statistical analysis

Statistical analysis was conducted with the SPSS18.0 software (SPSS Inc., Chicago, IL, USA). The differences among groups were analyzed using the chi-square test. Statistical significance was determined at *p* < 0.05.

## Results

### Sample information

A total of 1409 prenatal samples were analyzed, with the mean maternal age being 26.2 ± 3.3 years old and the mean gestational age being 18.3 ± 2.4 weeks. Chromosomal abnormalities were detected by karyotyping and BoBs assay, with 4 cases missed by karyotyping, 2 cases missed by BoBs assay, and 1403 cases successfully analyzed. As illustrated in Table 2, the 1403 successfully analyzed cases were grouped based on different high-risk factors (Table 2).

**Table 2:** Chromosomal abnormalities in different high-risk factors groups

High risk factors	Cases	Aneuploidies	Microdeletion/ microduplication	Other chromosomal abnormalities	Detection rate
Advanced maternal age	190	6	3	3	6.32%
High-risk NIPT results	163	65	3	0	41.72%
Abnormal ultrasound	134	8	6	4	13.43%
High-risk serological screening results	478	2	7	3	2.51%
Adverse pregnancy history	159	1	0	2	1.89%
Two or more types of high-risk factors	279	12	4	11	9.68%
Total	1403	94	23	23	9.98%

### Chromosomal abnormalities detected by karyotyping and BoBs assay

In this study, the success rates were 99.72% (1405/1409) for the karyotyping and 99.85% (1407/1409) for BoBs assay. Moreover, 140 cases of chromosomal abnormalities were identified by either karyotyping or BoBs assay, with a detection rate of 9.98% (140/1403) (Table 3). Among these cases, 116 cases of chromosomal abnormalities were detected by karyotyping, eliciting a detection rate of 8.27% (116/1403). On the other hand, 23 cases of chromosome microdeletion/microduplication and 1 case of mosaicism were detected by BoBs assay but missed by karyotyping (Table 3). Thus, a 1.71% (24/1403) incremental yield of the BoBs assay over karyotyping was observed.

**Table 3:** Chromosomal abnormalities detected by BoBs and karyotyping

Abnormality	Total number	Karyotyping	BoBs
Chromosomal aneuploidies	94	94	94
Trisomy 21	62	62	62
Trisomy 18	9	9	9
Trisomy 13	2	2	2
47,XXY	12	12	12
47,XXX	7	7	7
45,X	1	1	1
48,XXYY	1	1	1
Other chromosomal abnormalities	23	22	2
Inversion	12	12	0
Translocation	5	5	0
Mosaicism	6	5	2
Microdeletion/microduplication	23	0	23
Total	140	116	119

### Other chromosomal abnormalities detected by chromosome karyotyping and BoBs assay

**Fig. 1:**
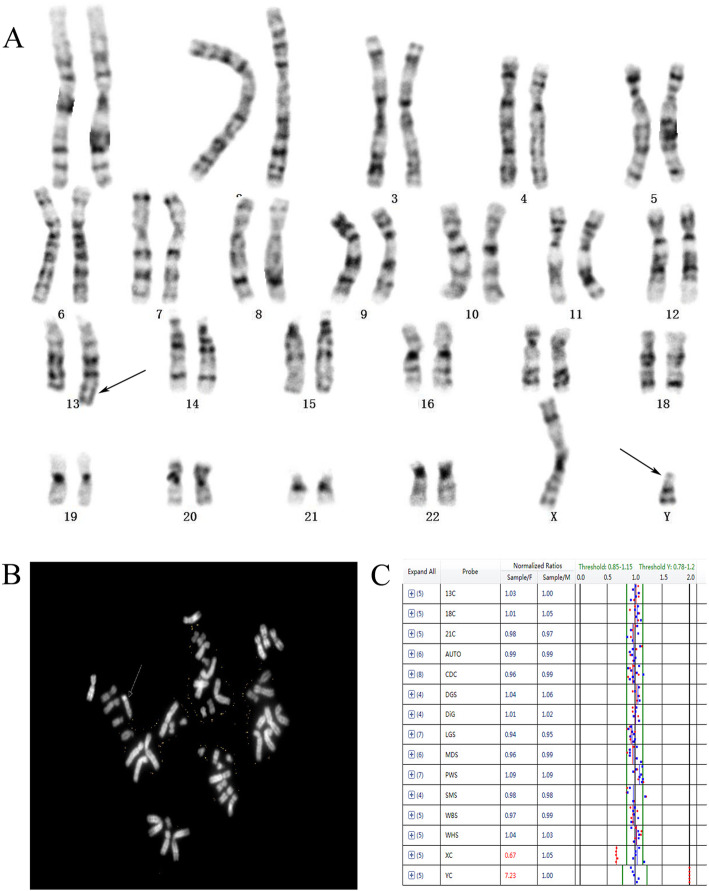
A balanced translocation case between chromosomes 13 and Y. **a** Karyotype analysis suspecting a translocation between chromosome 13 and the chromosome Y, and the arrows indicate the suspected translocation chromosomes. **b** C-banding was used to confirm the translocation, and the arrow indicates the translocation chromosome. **c** BoBs assay yielded normal results

**Fig. 2:**
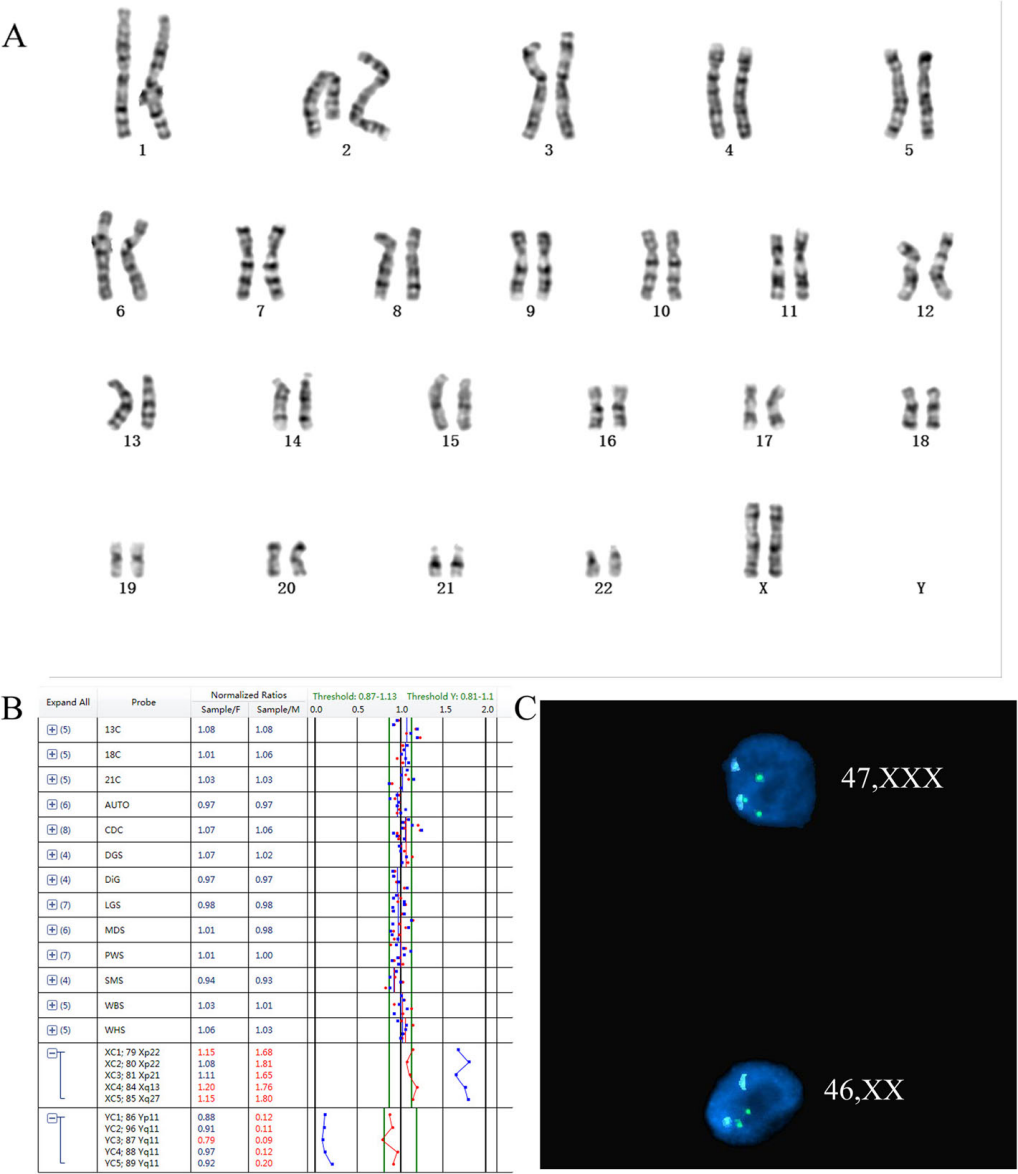
Chromosome mosaicism detected by BoBs assay and confirmed by FISH but missed by karyotyping. **a** Karyotype analysis result showed a normal female. **b** BoBs assay results revealed a gain of X chromosomes compared with normal males (Blue line), with the ratio being 1.5–2.0, which suggests an XX/XXX chromosome mosaicism. **c** The BoBs assay was verified by FISH. The green fluorescence represents the X chromosome, and the blue fluorescence represents chromosome 18, which serves as a control

Contrastly, 17 cases with structural abnormalities were detected by karyotyping but missed by BoBs assay, including 12 cases of chromosome inversion, 2 cases of Robertsonian translocation, and 3 cases of balanced translocation (Table 4) (Fig. 1). In this study, 6 cases of chromosome mosaicism were detected, among them 1 case was missed by karyotyping (Fig. 2) and 4 cases were missed by BoBs assay (Table 4).

**Table 4:** Other chromosomal abnormalities detected by karyotyping

Chromosomal abnormalities	FISH	BoBs	Cases
46,XN,inv.(9)(p11;q13)	/	normal	9
46,XY,inv.(Y)(p11.1q11.2)	/	normal	3
46,XN,inv.(9)(p11;q13),rob(14;21)(q10;q10)	/	normal	1
46,XN,rob(13;21)(q10;q10)	/	normal	1
46,XN,t(1;5)(p36;q13)	/	normal	1
46,XN,t(2;7)(p10;q10)	/	normal	1
46,XY, t(13;Y)(q11.23;q32)	/	normal	1
mos 45,XN,dic,t(5;14)(p15;p11.2[35]/ 45,XN,dic,t(8;14)(p32;q11.2)[15]	/	normal	1
mos 45,X[40]/47,XYY[3]/46,XY[10]	XO(70%)/XYY(20%) /XY (10%)	normal	1
mos 45,X[18]/47,XXX[6]/46,XX[76]	XO(22%)/XXX(2%)/XX(76%)	normal	1
mos 45,X[45]/46,XX[5]	XO(85%)/XX(15%)	XO	1
mos 47,XN,+ 18[30]/46,XN[20]	XN,+ 18 (80%)/XN (20%)	XN/XN,+ 18	1
46,XX	XX(97%)/XXX(3%)	XX/XXX	1
Total			23

### Microdeletion/microduplication detected by BoBs assay

**Fig. 3:**
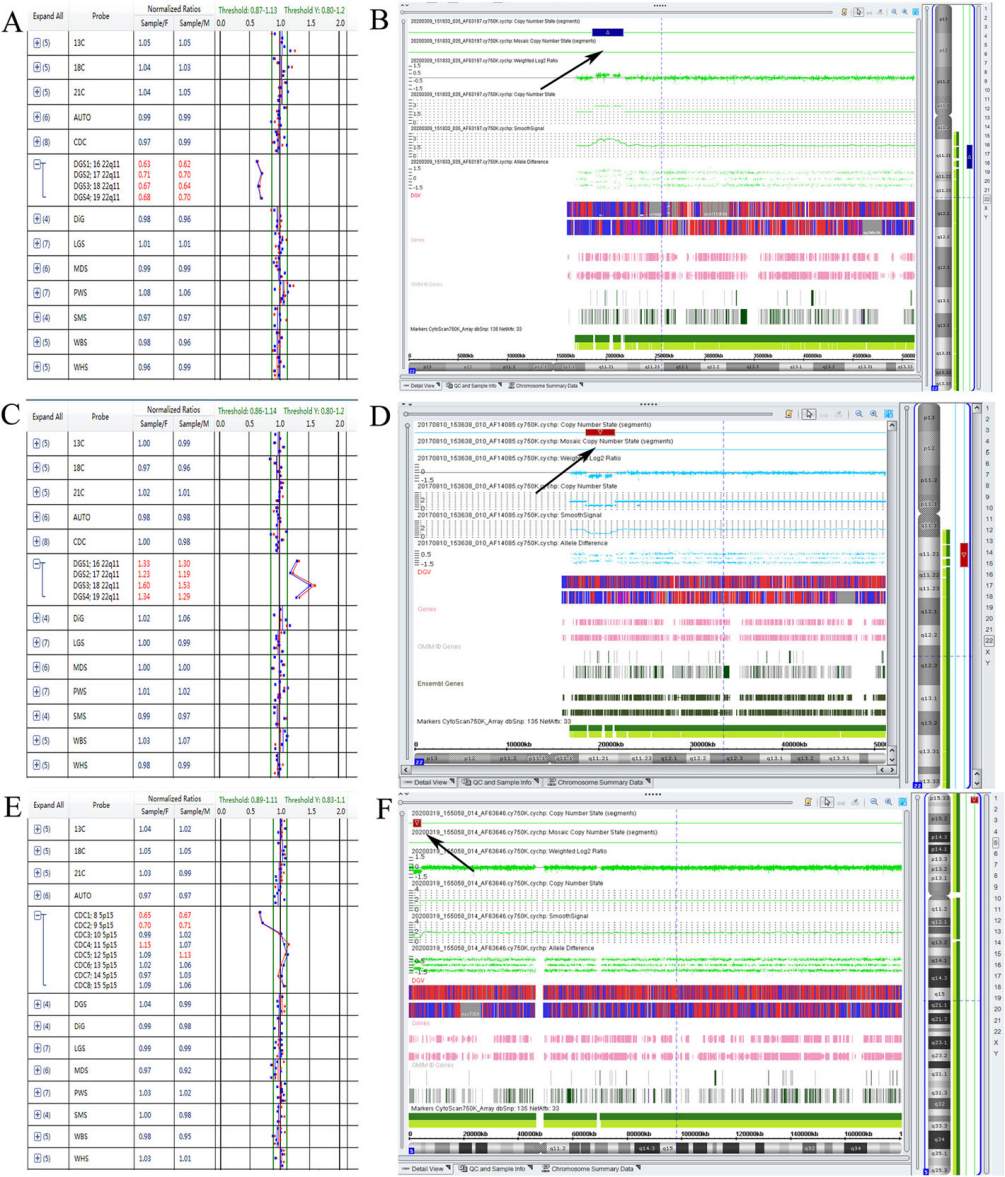
Microdeletions/microduplications detected by BoBs and confirmed by CMA. **a**, **b** BoBs assay revealed a 22q11.2 microduplication. SNP array verification was consistent with BoBs assay, and the arrow indicates the duplication region. **c**, **d** BoBs assay depicted a 22q11.2 microdeletion. SNP array verification was consistent with this finding, and the arrow points out the deletion region. **e**, **f** BoBs assay revealed a 5p15.32p15.33 microdeletion. SNP array verification was consistent with this finding, and the arrow indicates the deletion region

Moreover, 23 cases of microdeletions/microduplications were detected by BoBs assay (Table 5), including 2 cases of 22q11.2 microduplication, 2 cases of 22q11.2 microdeletion and 1 case of 5p15.32p15.33 microdeletion (Fig. 3), which were all confirmed by CMA and interpreted as pathogenic copy number variants (pCNVs). Additionally, 18 cases of sex chromosome microdeletion/microduplication were detected, including 13 cases of Xp22.31 microdeletion/microduplication, 3 cases of Yq11.221q11.222 microduplication, 1 case of Yp11.2 microduplication and 1 case of Xq27.3 microdeletion (Table 5). After genetic counseling, three families with Xp22.31 microdeletion/microduplication chose to terminate their pregnancies, including 2 fetuses with Xp22.31 microdeletions and 1 fetus with an Xp22.31 microduplication.

**Table 5:** Microdeletions/microduplications detected by BoBs assay

Karyotyping	Cases	BoBs	CMA	Pathogenicity	Pregnancy outcome
46,XN	2	22q11.2 microduplication	arr [hg19] 22q11.21(18,648,855-21,800,471)×3,3.1 Mb	pCNVs	Pregnancy termination
46,XN	2	22q11.2 microdeletion	arr [hg19] 22q11.21(18,648,855-21,800,471)×1,3.1 Mb	pCNVs	Pregnancy termination
46,XN	1	5p15.32p15.33 microdeletion	arr [hg19] 5p15.33p15.32(1,708,529-4,597,389)× 1,2.8 Mb	pCNVs	Pregnancy termination
46,XX	6	Xp22.31 microduplication	arr [hg19] Xp22.31(6,455,151-8,135,568)×3,1.6 Mb	VOUS	Normal pregnancy(5)/ Pregnancy termination(1)
46,XY	3	Xp22.31 microduplication	arr [hg19] Xp22.31(6,455,151-8,135,568)×2,1.6 Mb	VOUS	Normal pregnancy
46,XX	2	Xp22.31 microdeletion	arr [hg19] Xp22.31(6,455,151-8,135,568)×1,1.6 Mb	pCNVs	Normal pregnancy(1)/ Pregnancy termination(1)
46,XY	2	Xp22.31 microdeletion	arr [hg19] Xp22.31(6,455,151-8,135,568)×0,1.6 Mb	pCNVs	Normal pregnancy(1)/ Pregnancy termination(1)
46,XY	3	Yq11.221q11.222 microduplication	arr[hg19]Yq11.222(20,618,887-21,028,944) ×2,410.0 kb	bCNVs	Normal pregnancy
46,XX	1	Xq27.3 microdeletion	/	/	Normal pregnancy
46,XY	1	Yp11.2 microduplication	/	/	Normal pregnancy
Total	23				

*Abbreviations*: *pCNVs* Pathogenic copy number variants, *bCNVs* Benign copy number variants

### Chromosomal abnormalities detection rates across the groups

Significant differences in detection rates of chromosomal abnormality were observed across groups with different high-risk factors (*p* < 0.001). As delineated in Table 2, the high-risk NIPT results group demonstrated the highest detection rate (41.72%), followed by the ultrasound abnormality group (13.43%) and the group with two or more types of high-risk factors (9.68%) (Table 2). The high-risk serological screening results (1.89%) and adverse pregnancy history (2.51%) groups elicited the lowest detection rates, providing valuable data for prenatal diagnosis with the combined use of BoBs assay and karyotyping.

## Discussion

Chromosomal abnormalities are among the primary reasons for birth defects, with trisomy 21, trisomy 18, trisomy 13 and sex chromosome aneuploidies being the most prevalence entities [9]. Karyotype analysis of chromosomes from the amniotic fluid is the “gold standard” for chromosomal abnormality detection, whereas the BoBs assay can rapidly detect common aneuploidies and 9 common chromosome microdeletion syndromes without the need for cell culture. In this study, our initial aim was to explore the application value of BoBs in 1409 pregnant women admitted at Quanzhou women’s and children’s hospital and to identify differences in abnormality detection rates between different high-risk factor groups using a combination of BoBs assay and karyotyping.

In this study, 140 cases of chromosomal abnormalities were identified by the two detection technologies, with a detection rate of 9.98%, which was similar to previous studies [10, 11]. The two detection technologies yielded exactly the same diagnostic outcomes in the detection of common aneuploidies, and no false-positive or false-negative results were observed. Moreover, 17 cases of chromosome rearrangement were detected by karyotyping but missed by BoBs assay. Among these rearrangements, a suspected translocation of chromosome 13 and Y was confirmed by C-banding (Fig. 1). Furthermore, 6 cases of chromosome mosaicism were detected, and 4 cases of chromosome mosaicism were missed by BoBs assay. One of the cases was a balanced translocation mosaicism, which was missed by BoBs assay. Another case missed by BoBs assay was a mos 45, X[45]/46, XX[5], which may be due to this technology’s intrinsic limitation for detecting mosaicism in various targeted regions, with detection rates usually ranging from 20 to 40% [12]. According to the research, karyotype analysis has a higher detection rate of mosaicism compared with BoBs. Nonetheless, one case of XX/XXX chromosome mosaicism was detected by BoBs assay but missed by karyotyping, and further FISH verification results were consistent with BoBs assay. Therefore, combining karyotyping with BoBs assay may allow for more effective detection of chromosome mosaicism than is achieved using each method alone.

In this study, 23 cases of chromosome microdeletions/microduplications were detected by BoBs assay, including 2 cases of 22q11.2 microduplication syndrome with a 3.1-Mb duplication. Previous studies have reported that 22q11.2 duplications can lead to a clinical phenotype in some subjects, while others exhibit a normal phenotype, indicating incomplete penetrance of the duplication [13]. In some cases, the pregnant women and their families eventually opted for pregnancy termination after clinical consultation. Besides, two cases of 22q11.2microdeletion were depicted, with a 3.1-Mb size deletion involving the region of DiGeorge syndrome (DGS) and Velo-Cardio-Facial syndrome (VCFS), which was interpreted as pCNVs. The families chose pregnancy termination due to the detection of fetal cardiac abnormalities and fetal growth restriction. Moreover, one case with a 5p15.2p15.3 microdeletion was detected, delineating a 2.8-Mb size deletion but did not involve the critical region for Cri-du-Chat syndrome. In this case, 6 genes were covered by the deletion, the *NDUFS6* gene, which is associated with mitochondrial complex I deficiency disease [14]. Further parental investigation showed that the 5p15.2p15.3 microdeletion was inherited from his mother who exhibited a clinical phenotype with mild mental retardation; the family chose to terminate the pregnancy after clinical consultation. The incidence rate of microdeletion/microduplication syndromes (0.36%, 5/1403) in our study was lower than the incidence rates reported in some studies [11, 15], but similar to those reported in other studies [8, 16, 17]. Additionally, 18 cases of sex chromosome microdeletion/microduplication were detected by BoBs assay. Among these cases, microdeletions and microduplications frequently occurred at the Xp22.3 locus. Deletions or mutations of the Xp22.31 locus are associated with X-linked ichthyosis (OMIM 308100, XLI), which is a dermatologic disorder with characteristic dry and scaly skin due to a deficiency of the enzyme steroid sulfatase [18, 19]. In this study, 10 pregnant women with Xp22.31 microdeletion and microduplication chose to continue their pregnancy, and the pregnancies outcome results were normal.

Significant differences in chromosomal abnormality detection rates were observed among the groups with different high-risk factors. Of these groups, the high-risk NIPT results group and ultrasound abnormality group showed high detection rates of chromosomal abnormalities, consistent with other studies [20–22]. Hence, it is believed that BoBs assay is a reliable prenatal diagnostic tool for the rapid detection of common aneuploidies and microdeletion/microduplication syndromes. Nevertheless, BoBs assay is unable to detect deletions and duplications in chromosomal regions not covered by the assay, point mutations, balanced rearrangements (inversions and translocations), ploidy changes, methylations, and UPD (uniparental disomy) [16, 23]. Thence, CMA can be used to screen for CNVs of the whole genome. In fact, combined use of CMA and karyotyping can effectively improve the detection rate of chromosomal mosaicism, balanced translocations and inversions. Likewise, CMA is recommended as a first-line prenatal diagnostic tool for fetal ultrasound structural abnormalities [3]. A recent study has shown that CMA should be performed in cases with fetal structural anomalies and/or stillbirth, and replaces the need for fetal karyotyping in these cases [24]. Moreover, the study conducted by Stosic et al. suggests that all women undergoing invasive testing for routine indications should be subjected to chromosomal microarray [25]. It is believed that with continuous application of CMA in conjunction with the available databases, CMA detection technology will replace BoBs assay and karyotyping for prenatal diagnosis in the future.

## Conclusions

This study first investigated the application value of BoBs assay for prenatal diagnosis in Quanzhou, which provides baseline data regarding the combined application of BoBs assay and karyotyping for prenatal diagnosis of chromosomal abnormalities. In this study, we further proved that BoBs assay is a rapid and reliable detection technology for the prenatal diagnosis of common aneuploidies and microdeletion/microduplication syndromes, while its ability to detect chromosomal mosaicisms is limited. Furthermore, the combined use of BoBs assay and karyotyping in prenatal diagnosis may allow for a more effective detection of chromosomal abnormalities.

## Supplementary Information

**Additional file 1.**

## Data Availability

All data generated or analysed during this study are included in this published article and its supplementary information files.

## Abbreviations

BoBs: BACs-on-Beads
CMA: Chromosome microarray analysis
NIPT: Non-invasive prenatal testing
FISH: Fluorescence in situ hybridization
CNVs: Copy number variants
OMIM: Online Mendelian Inheritance in Man
